# Platinum thin film–based matrix-enhanced surface-assisted laser desorption/ionization mass spectrometry imaging for improved ion yields of adrenocortical steroids

**DOI:** 10.1007/s44211-026-00935-9

**Published:** 2026-06-25

**Authors:** Riko Takata, Shigehiro Karashima, Kokoro Okawa, Akio Miyazato, Fumitake Usui-Kawanishi, Reina Nakanishi, Tatsuya Murakami, Atsushi Hashimoto, Hiroshi Kawasaki, Issey Osaka

**Affiliations:** 1 Department of Pharmaceutical Engineering, Faculty of Engineering, Toyama Prefectural University, 5180 Kurokawa, Imizu-City, Toyama, 939-0398 Japan; 2 Faculty of Textile Science and Technology, Shinshu University, Ueda, Nagano, 386–8567 Japan; 3 Institute of Liberal Arts and Science, Kanazawa University, Kanazawa, Ishikawa 920-1192 Japan; 4 Center for Nano Materials and Technology, Japan Advanced Institute of Science and Technology, Nomi, Ishikawa 923–1292 Japan; 5 Faculty of Transdisciplinary Sciences, Institute of Philosophy in Interdisciplinary Sciences, Kanazawa University, Kanazawa, Ishikawa 920-1192 Japan; 6 Department of Medical Neuroscience, Graduate School of Medical Sciences, Kanazawa University, Kanazawa, Ishikawa 920-8640 Japan

**Keywords:** SALDI, ME-SALDI, MALDI, MS imaging, Adrenocortical steroids

## Abstract

Matrix-assisted laser desorption/ionization mass spectrometry (MALDI/MS) imaging enables spatially resolved molecular analysis; however, its sensitivity depends strongly on matrix uniformity and ionization efficiency, which pose challenges for low-abundance compounds such as steroid hormones. In this study, we developed a platinum (Pt) thin film–based surface-assisted laser desorption/ionization mass spectrometry (SALDI/MS) and matrix-enhanced SALDI/MS (ME-SALDI/MS) method to increase ion yields and improve steroid imaging with minimal sample pretreatment. Using sputter-deposited Pt films, 20 endogenous steroids were comprehensively detected without chemical derivatization. Furthermore, derivatized steroids analyzed by ME-SALDI/MS exhibited significantly higher ion yields than those obtained by conventional MALDI/MS or SALDI/MS, demonstrating a synergistic enhancement of desorption and ionization efficiency. ME-SALDI/MS imaging of porcine adrenal gland tissue enabled visualization of multiple derivatized steroids with high ion yields—including progesterone, corticosterone, and cortisol—reflecting their biosynthetic localization across cortical zones. Collectively, these results highlight the potential of Pt thin film–based SALDI and ME-SALDI/MS as robust tools that provide high ion yields for imaging of steroids and other biomolecules with inherently low desorption and ionization efficiency.

**Graphical abstract:**

**Figure:**
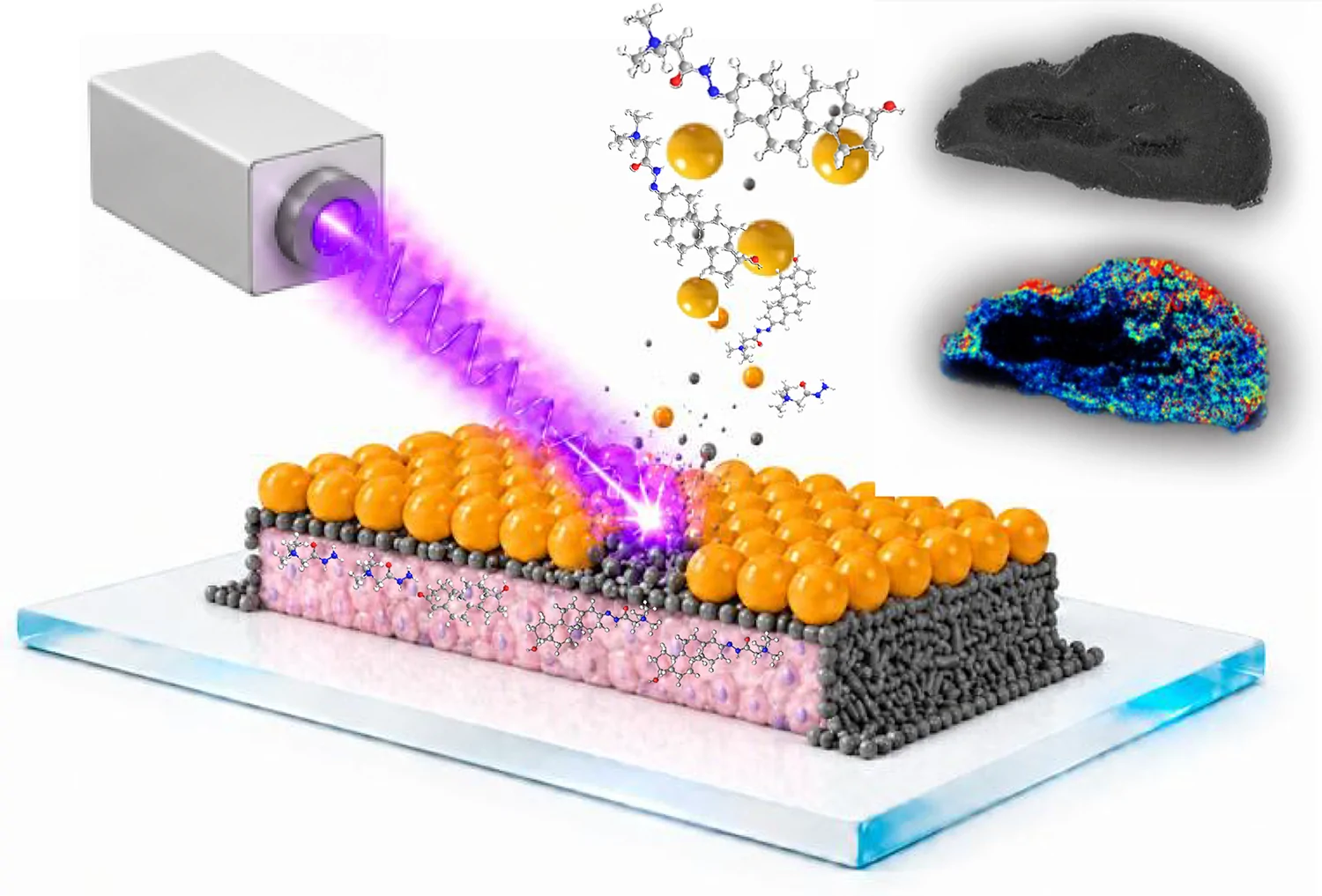

## Introduction

Matrix-assisted laser desorption/ionization mass spectrometry (MALDI/MS) imaging is a powerful analytical technique that enables simultaneous acquisition of mass and spatial information of compounds distributed on a sample surface. MALDI/MS has been widely applied across research fields, including molecular biology, food science, and medicine [1–3]. For MALDI/MS imaging, uniform coating of matrices and derivatization reagents on the tissue surface is essential for reproducible ionization. Accordingly, a range of coating approaches has been developed, including commonly used methods such as air-spray deposition [4] and vacuum deposition [5]. Because detection sensitivity in MALDI depends strongly on matrix selection, optimization of matrix species and coating conditions remains critical for reliable MALDI/MS analysis.

Surface-assisted laser desorption/ionization mass spectrometry (SALDI/MS) has recently been investigated as a complementary technique to MALDI/MS [6, 7]. SALDI is also a soft ionization method, but it uses UV-absorbing materials—such as metal nanoparticles or metal films—as ionization-assisting substrates instead of organic matrices [8]. When non-ionizable inorganic materials are used, background noise in the low *m/z* range is significantly reduced relative to MALDI, facilitating detection of low-molecular-weight compounds [9]. For example, diacylglycerols, which are difficult to ionize by MALDI, have been successfully detected using metal-film-based SALDI/MS, demonstrating its distinct ionization capabilities [10, 11]. Metal films are typically fabricated by sputter deposition, enabling the formation of uniform solvent-free films without requiring matrix crystallization control or advanced coating skills. Furthermore, to enhance the ionization efficiency in SALDI, matrix-enhanced surface-assisted laser desorption/ionization mass spectrometry (ME-SALDI/MS) has been proposed, in which the MALDI matrix is deposited onto the ionization-assisting material [12, 13]. This approach enables highly sensitive detection of various drugs and endogenous metabolites [13], demonstrating the broad applicability of ME-SALDI/MS. However, despite recent methodological advances in SALDI and ME-SALDI/MS, their application to steroid hormone analysis remains limited, particularly in the context of tissue imaging. In this field, conventional MALDI/MS analyses still require multi-step derivatization and matrix-coating procedures to achieve adequate sensitivity and ionization efficiency [14]. To the best of our knowledge, this study provides the first evidence that a sputtered metal thin-film substrate combined with minimal matrix addition enables high-sensitivity imaging of steroid hormones, improving ionization efficiency while simplifying sample pretreatment.

In recent years, endocrine steroid hormones have been analyzed by MALDI/MS imaging. Steroids are biosynthesized from cholesterol and are produced mainly in the ovaries, testes, and adrenal cortex [15]. The adrenal cortex has a three-layered structure consisting of the zona glomerulosa, zona fasciculata, and zona reticularis, in which distinct classes of steroids are synthesized. Spatially resolved analysis of trace-level steroids within this zonal structure is crucial for elucidating endocrine functions and disease mechanisms [16]. However, steroid hormones are present at trace levels and exhibit extremely low desorption/ionization efficiency in MALDI. Therefore, chemical derivatization is generally required to improve detection sensitivity in MALDI/MS imaging. For example, Girard reagents have enabled detection of several steroids, including testosterone, corticosterone, and progesterone [17–19]. However, these detections have primarily been reported under conditions in which target steroids are relatively abundant, such as in human organs or model animals. Accordingly, improved detection sensitivity is increasingly important for biological and pathological investigations, in which trace-level variations and decreases in specific constituents must be detected.

In this study, we applied SALDI/MS and ME-SALDI/MS using platinum (Pt) thin films for the detection of steroid hormones with high ion intensities. SALDI/MS with Pt films enabled the detection of steroid hormones with minimal manual pretreatment. In addition, ME-SALDI/MS of derivatized steroids provided higher ion yields than conventional methods. We evaluated analytical performance and performed MS imaging of porcine adrenal gland tissue sections to assess the practical utility of the proposed method. This work presents a Pt thin film–based ME-SALDI platform for steroid imaging and, to the best of our knowledge, provides the first demonstration of adrenal steroids visualization with high ion yields using a metal-film-assisted ionization strategy.

## Experimental

### Materials

α-Cyano-4-hydroxycinnamic acid (CHCA), estrone, androstenedione, testosterone, dehydroepiandrosterone (DHEA), dihydrotestosterone (DHT), adrenosterone, 11-ketotestosterone, pregnenolone, 11-deoxycorticosterone (11-DOC), 17α-hydroxyprogesterone (17α-OHP), 21-deoxycortisol, aldosterone, cortisone, and 18-hydroxycorticosterone (18-OHB) were purchased from Sigma-Aldrich (St. Louis, MO, USA). Corticosterone, Girard’s Reagent T (GirT), and trifluoroacetic acid (TFA) were obtained from Tokyo Chemical Industry Co., Ltd. (Tokyo, Japan). 16α-Hydroxyprogesterone (16α-OHP) and 18-oxocortisol were purchased from Toronto Research Chemicals (Toronto, Ontario, Canada). 11-Deoxycortisol and progesterone were obtained from Makor Chemicals Ltd. (Jerusalem, Israel). 11β-Hydroxyprogesterone (11β-OHP) was purchased from ChemScene LLC (Monmouth Junction, NJ, USA). Cortisol, Mayer’s hematoxylin solution, eosin, HPLC-grade acetonitrile (AcN), methanol (MeOH), acetic acid, and 20% formalin solution were purchased from FUJIFILM Wako Pure Chemical Corporation (Osaka, Japan). OCT Compound for rapid embedding of fresh tissue specimens for frozen sectioning using a cryostat was obtained from Sakura Finetek Japan Co., Ltd. (Tokyo, Japan). Ultrapure water was prepared using a Milli-Q Integral system (Merck Millipore, Burlington, MA, USA). The Pt target used for sputter deposition was purchased from Furuuchi Chemical Corporation (Tokyo, Japan).

### Tissue sectioning and mounting

Adrenal gland tissue from female pigs was purchased from Toyama Meat Inspection Center Co., Ltd. (Toyama, Japan). The tissue samples were rapidly frozen in liquid nitrogen immediately after collection.

Tissue samples were embedded in O.C.T. Compound for hematoxylin and eosin (HE) staining. Frozen sections were prepared at − 20 °C using a Leica CM1860 UV cryostat (Leica Biosystems, Germany). For MS imaging, the tissue was sectioned at 20 µm and mounted on indium tin oxide (ITO)-coated glass slides (Bruker Daltonics Inc., Bremen, Germany). For H&E staining, the tissue was sectioned at 10 µm and mounted on MAS-coated glass slides (Matsunami Glass, Osaka, Japan).

### Histological staining

The frozen tissue sections were fixed in 20% formalin solution for 30 s, stained with hematoxylin for 5 min, and washed under running tap water for 5 min. The sections were then rinsed sequentially in ultrapure water and 90% ethanol, followed by counterstaining with eosin for 1 min. The sections were subsequently dehydrated in ethanol, cleared in xylene, and mounted with mounting medium. The stained tissue sections were examined using an IXplore Pro microscope (Evident Scientific, Tokyo, Japan).

### Sample preparation

The CHCA matrix solution for MALDI was prepared at 10 mg/mL in a water/acetonitrile mixture (1/1, v/v). Standard steroid solutions were prepared at 10 µg/mL in water/methanol (1/1, v/v). The GirT solution used for derivatization was prepared at 10 µg/mL in methanol containing 20% acetic acid. For tissue imaging experiments, a 10 mg/mL GirT solution was prepared in methanol containing 20% acetic acid for on-tissue derivatization.

For MALDI/MS measurements of derivatized standard steroids, 1 µL each of the standard steroid solution, derivatization solution, and CHCA solution were spotted sequentially on the plate and dried at room temperature. For SALDI/MS measurements of standard steroids, 1 µL of the standard steroid solution was spotted and air-dried, followed by sputter deposition of a Pt thin film onto the dried sample. For ME-SALDI/MS measurements of standard steroids, 1 µL of the standard steroid solution was spotted and dried, a Pt thin film was fabricated by sputtering on the dried residue, and 1 µL of the CHCA solution was then spotted and air-dried.

For MALDI/MS and ME-SALDI/MS imaging, the CHCA matrix solution was prepared at 10 mg/mL in a water/acetonitrile mixture (1/1, v/v) containing 0.1% TFA. After deposition of the Pt thin film directly onto the sample, CHCA was applied to the Pt-coated surface by spray coating. The CHCA solution was sprayed onto the sample surface using an airbrush (Procon Boy WA Double Action Platinum 0.2 mm, Mr. Hobby, Japan). The matrix solution was first sprayed using an airbrush positioned 25 cm from the plate to deposit 2 mL, followed by an additional 5 mL applied from a distance of 20 cm.

### On-tissue chemical derivatization

For derivatization with GirT, a 10 mg/mL GirT solution was prepared in methanol containing 20% acetic acid. The solution was sprayed onto the sample surface using an airbrush (Procon Boy WA Double Action Platinum 0.2 mm, Mr. Hobby, Japan). Spraying was performed with the airbrush positioned 20 cm from the plate, and a total of 0.5 mL was deposited. To humidify the chamber, a laboratory wiper (Kimwipe, Nippon Paper Crecia Co., Ltd., Tokyo, Japan) prewetted with 500 µL of water/methanol (2:8, v/v) was placed in a slide glass chamber (82 × 29 × 18 mm) in advance. After spraying, the ITO-coated glass slides were placed in the sealed humidified chamber and incubated at 40 °C for 20 min, followed by drying at 40 °C for 10 min.

### Mass spectrometry

MALDI/MS, SALDI/MS, and MS imaging of porcine adrenal gland tissue sections were performed using a time-of-flight mass spectrometer (TOFMS, JMS-S3000 SpiralTOF-plus, JEOL Ltd., Tokyo, Japan) equipped with an Nd:YLF laser (349 nm). Measurements were performed in Spiral mode under the following conditions: laser power, 35–60%; delay time, 45 ns; acceleration voltage, 20 kV; and pulsed laser frequency, 500 Hz. These parameters were optimized for each target analyte. MS imaging data were acquired at a spatial resolution of 100 µm in positive ion mode. Each mass spectrum was obtained by accumulating 50 laser shots. For each measurement, the 50-shot acquisition was repeated five times, and the ion intensities were averaged. Data analysis was performed using msTornado™ Analysis software (JEOL Ltd., Tokyo, Japan). Accurate *m/z* calibration was performed using 1.0 mg/mL methanolic solutions of polyethylene glycol (PEG) with average molecular weights of 200 and 400 (Sigma-Aldrich), respectively.

### Preparation of platinum thin film

Pt thin films were deposited using a magnetron sputtering apparatus (MSP-1S, Vacuum Device Inc., Ibaraki, Japan) under the following conditions: discharge current, 40 mA; target-to-sample distance, 35 mm; and deposition time, 9 s. The thickness of the Pt film was optimized for the present study [12]. Films that are too thin provide insufficient desorption/ionization efficiency, whereas excessively thick films hinder desorption. The resulting Pt layer on the sample surface was approximately 3 nm.

## Results and discussion

### Comparison of MALDI, SALDI, and ME-SALDI/MS

**Fig. 1:**
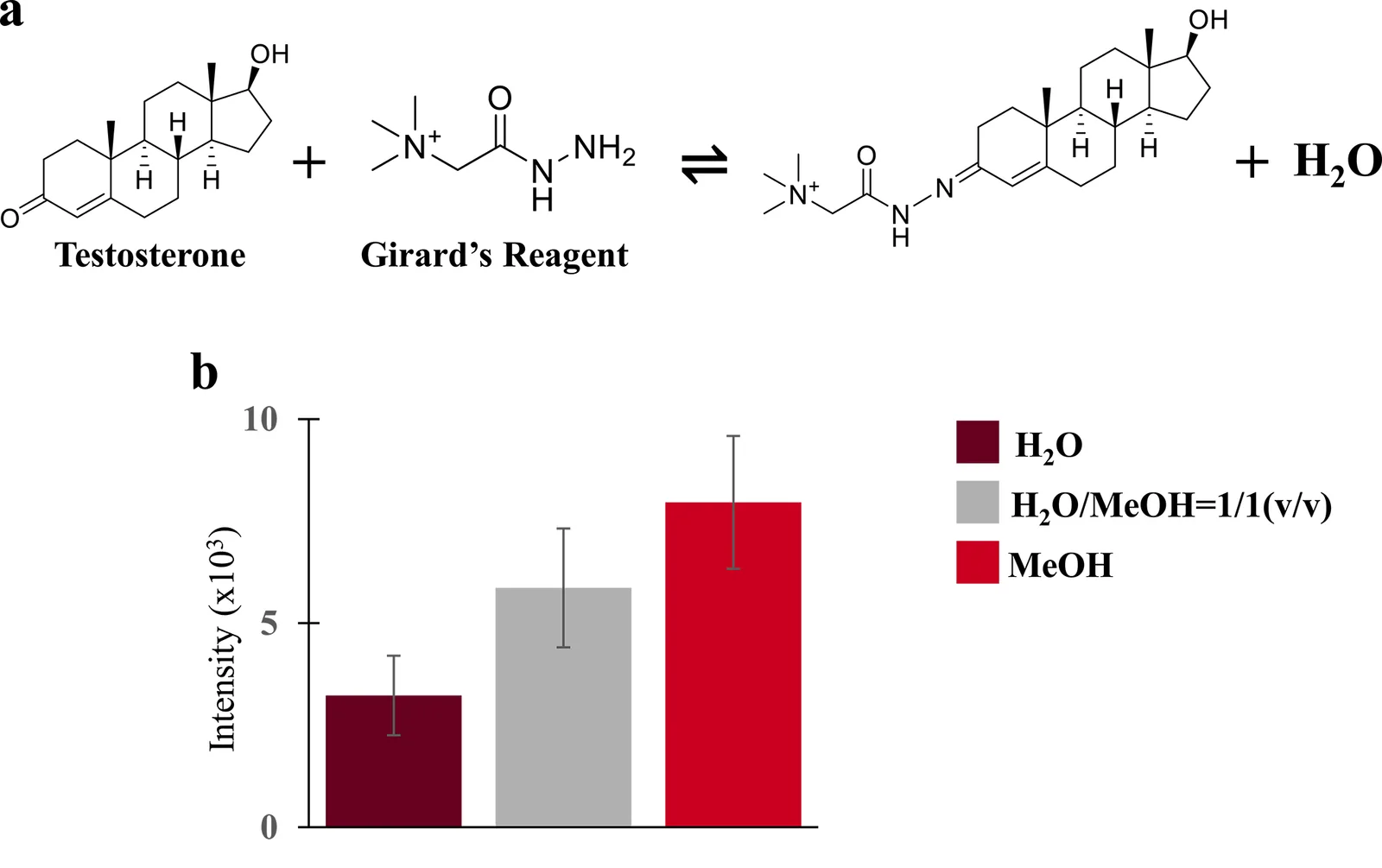
**a** Derivatization of testosterone with GirT. **b** Ion intensities of GirT derivatized testosterone in MALDI/MS. GirT was dissolved in H_2_O, H_2_O/MeOH = 1/1, or MeOH for derivatization; testosterone was derivatized with GirT under each condition. Error bars indicate standard deviation (n = 5)

The derivatization conditions for GirT were first optimized. GirT solutions were prepared in methanol, water, or a 1/1 (v/v) water/methanol mixture, and testosterone was derivatized and analyzed by MALDI/MS. [M]^+^ ions corresponding to GirT-hydrazone derivatives were observed, with methanol yielding the highest signal intensity owing to its superior solubility for steroids (Fig. 1). Although previous studies employed water/methanol mixtures [20, 21], this reaction proceeds via dehydration; therefore, the presence of water likely reduces reaction efficiency. Based on these results, methanol was selected as the optimal solvent for GirT derivatization. Collectively, these findings indicate that solvent composition must be carefully controlled to ensure efficient GirT derivatization and to maximize ionization efficiency in subsequent mass spectrometric analysis.

**Fig. 2:**
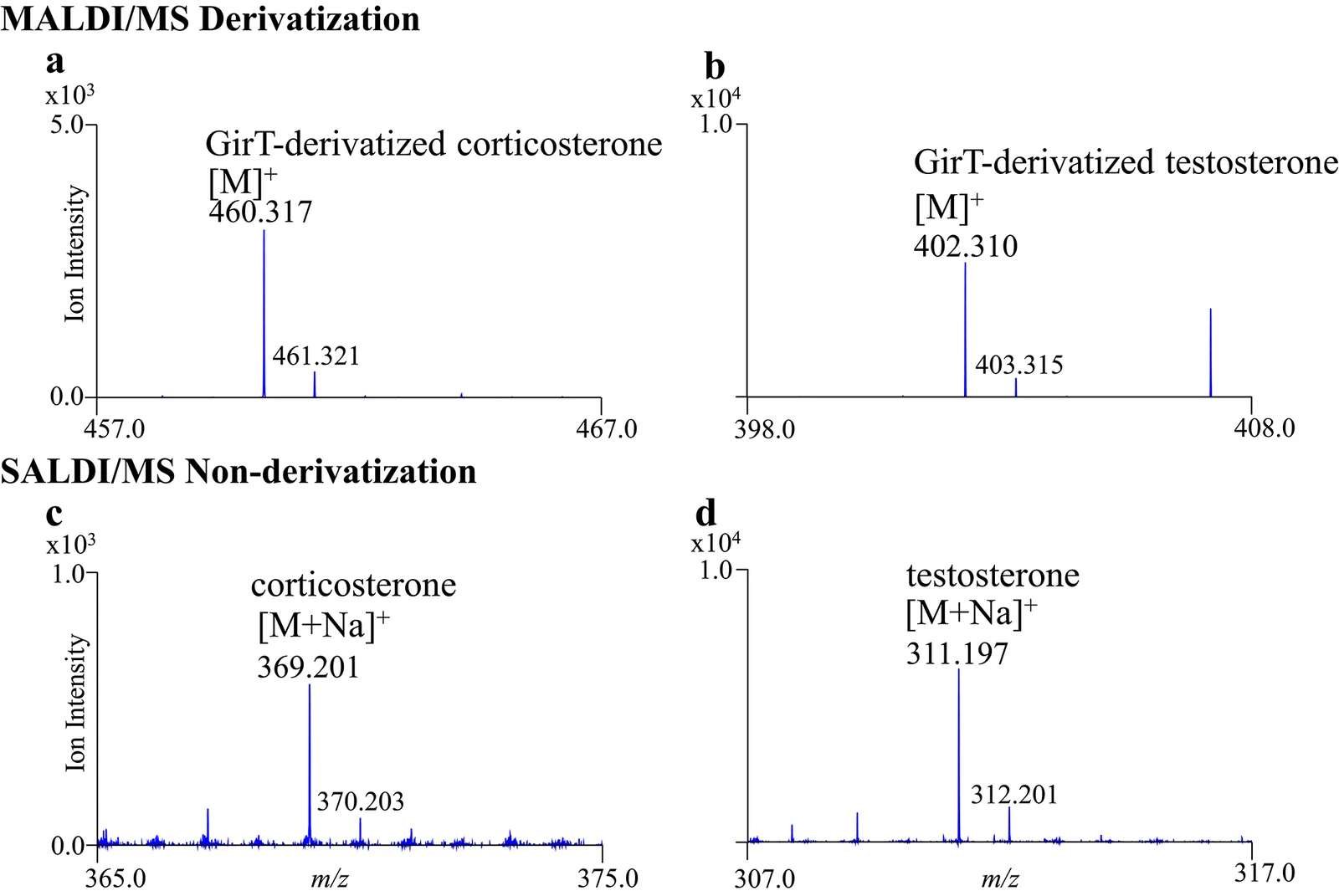
Mass spectra of GirT derivatized corticosterone and testosterone in MALDI/MS, and non-derivatized corticosterone and testosterone in SALDI/MS. **a** GirT-derivatized corticosterone in MALDI/MS **b** GirT-derivatized testosterone in MALDI/MS **c** non-derivatized corticosterone in SALDI/MS **d** non-derivatized testosterone in SALDI/MS

To compare ionization behavior, the MALDI/MS spectra of GirT derivatized corticosterone and testosterone (Fig. 2a,b) and the SALDI/MS spectra of non-derivatized corticosterone and testosterone (Fig. 2c,d) were analyzed. In SALDI/MS using a 3-nm Pt thin film deposited by sputtering, [M + Na]^+^ ions were directly detected without derivatization. Corticosterone showed a higher signal intensity in MALDI/MS, whereas testosterone exhibited a stronger response in SALDI/MS, indicating that the optimal ionization method varies substantially depending on the physical properties of steroids. This comparison further indicates that ionization behavior in laser desorption/ionization mass spectrometry is not uniform among steroid species but depends on molecular structure and physicochemical properties. Accordingly, selecting an appropriate ionization strategy—MALDI, SALDI, or a hybrid approach—is essential for comprehensive and unbiased steroid profiling.

Subsequently, ion yields were evaluated for 20 standard steroids. Ion intensities obtained by MALDI/MS, SALDI/MS, and ME-SALDI/MS for GirT derivatized steroids were compared with those obtained by SALDI/MS and ME-SALDI/MS for non-derivatized steroids. Non-derivatized steroids could not be directly detected by MALDI/MS and were observed only after GirT derivatization. GirT derivatized products were successfully obtained for estrone, androstenedione, testosterone, DHEA, DHT, adrenosterone, 11-ketotestosterone, progesterone, 11β-OHP, 11-DOC, 16α-OHP, 17α-OHP, 11-deoxycortisol, 21-deoxycortisol, corticosterone, aldosterone, cortisone, 18-OHB, cortisol, and 18-oxocortisol. The observed *m/z* values of the [M]^+^ ions were 384.264, 400.293, 402.310, 402.311, 404.325, 414.274, 416.292, 428.326, 444.325, 444.323, 444.324, 444.323, 460.317, 460.316, 460.317, 474.297, 474.297, 476.310, 476.312, and 490.290, respectively. The theoretical exact masses were 384.265, 400.296, 402.312, 402.312, 404.327, 414.275, 416.291, 428.327, 444.322, 444.322, 444.322, 444.322, 460.317, 460.317, 460.317, 474.296, 474.296, 476.312, 476.312, and 490.291, respectively. The mass errors were within 0.005 Da, demonstrating excellent mass accuracy (Table 1). Non-derivatized steroid ions were successfully obtained for estrone, androstenedione, testosterone, DHEA, DHT, adrenosterone, 11-ketotestosterone, progesterone, 11β-OHP, 11-DOC, 16α-OHP, 17α-OHP, 11-deoxycortisol, 21-deoxycortisol, corticosterone, aldosterone, cortisone, 18-OHB, cortisol, and 18-oxocortisol. The observed *m/z* values of the [M + Na]^+^ ions were 293.151, 309.181, 311.197, 311.199, 313.213, 323.160, 325.177, 337.215, 353.204, 353.213, 353.212, 353.208, 369.201, 369.204, 369.208, 383.186, 383.184, 385.201, 385.199, and 399.179, respectively. The theoretical exact masses were 293.151, 309.183, 311.198, 311.198, 313.214, 323.162, 325.177, 337.214, 353.209, 353.209, 353.209, 353.209, 369.204, 369.204, 369.204, 383.183, 383.183, 385.199, 385.199, and 399.178, respectively. The mass errors were within 0.005 Da, demonstrating excellent mass accuracy (Table 1). These results clearly demonstrate the robustness and precision of the optimized analytical workflow, confirming that GirT derivatization combined with MALDI or ME-SALDI provides reliable mass accuracy across a broad range of steroid compounds. This performance supports application of the developed method to complex biological samples.

**Table 1:** Theoretical exact masses and observed *m/z* values of non-derivatized and derivatized steroid ions

Compounds	Non-derivatized steroid ion, [M + Na] +			Derivatized steroid ion,[M] +			
	Theoretical exact mass	Observed m/z	Observed *m/z*	Theoretical exact mass	Observed m/z	Observed *m/z*	Observed *m/z*
		SALDI/MS	ME-SALDI/MS		SALDI/MS	ME-SALDI/MS	MALDI/MS
Estrone	293.151	293.151	293.146	384.265	384.265	384.260	384.264
Androstenedione	309.183	309.181	309.182	400.296	400.296	400.292	400.293
Testosterone	311.198	311.197	311.196	402.312	402.313	402.311	402.310
DHEA	311.198	311.199	311.194	402.312	402.312	402.315	402.311
DHT	313.214	313.213	313.209	404.327	404.330	404.332	404.325
Adrenosterone	323.162	323.160	323.157	414.275	414.275	414.272	414.274
11-ketoteststerone	325.177	325.177	325.172	416.291	416.293	416.291	416.292
Progesterone	337.214	337.215	337.214	428.327	428.326	428.331	428.326
11β-OHP	353.209	353.204	353.210	444.322	444.324	444.323	444.325
11-DOC	353.209	353.213	353.208	444.322	444.321	444.327	444.323
16α-OHP	353.209	353.212	353.211	444.322	444.321	444.319	444.324
17α-OHP	353.209	353.208	353.207	444.322	444.323	444.322	444.323
11-deoxycortisol	369.204	369.201	369.204	460.317	460.316	460.322	460.317
21-deoxycortisol	369.204	369.204	369.201	460.317	460.316	460.314	460.316
Corticosterone	369.204	369.208	369.203	460.317	460.319	460.318	460.317
Aldosterone	383.183	383.186	383.178	474.296	474.296	474.293	474.297
Cortisone	383.183	383.184	383.180	474.296	474.297	474.297	474.297
18-OHB	385.199	385.201	385.196	476.312	476.311	476.310	476.310
Cortisol	385.199	385.199	385.197	476.312	476.312	476.311	476.312
18-oxocortisol	399.178	399.179	399.178	490.291	490.289	490.296	490.290

Collectively, these findings indicate that the optimal ionization technique varies significantly among steroids. Non-derivatized neutral steroids are difficult to desorb and ionize by MALDI because they lack proton-donor or -acceptor functional groups; however, SALDI enabled their desorption and ionization. Previous studies have reported that SALDI can ionize neutral lipids such as triacylglycerols and hexosylceramides, which are rarely detected by MALDI [22, 26]. Although the detailed ionization mechanism of SALDI remains unclear, the physicochemical properties of the ionization-assisting materials, including composition and morphology, strongly influence ionization efficiency. Pt, which is chemically stable against oxidation, has a high melting point, high crystallinity, and low thermal conductivity. These properties likely promote local heating under laser irradiation, thereby enhancing desorption efficiency [23]. In contrast, in MALDI, a portion of the laser energy is consumed by sublimation of the organic matrix, which may reduce effective energy transfer to the analyte. This difference in energy transfer may account for the improved ionization of steroids observed in SALDI/MS.

**Fig. 3:**
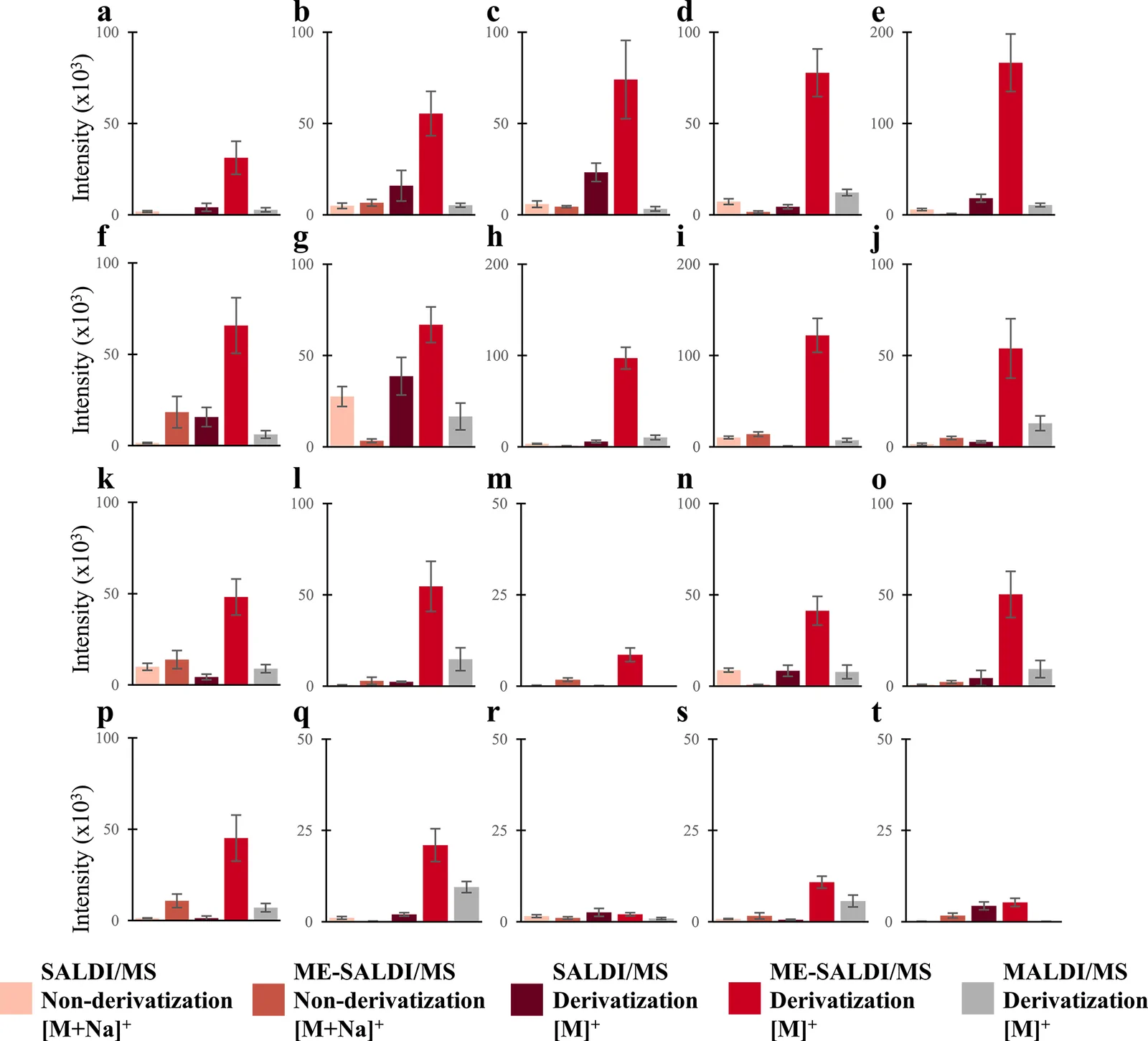
Comparison of ion intensities between SALDI/MS and ME-SALDI/MS for non-derivatized and derivatized steroids, and MALDI/MS for derivatized steroids. **a** estrone, **b** androstenedione, **c** testosterone, **d** DHEA, **e** DHT, **f** adrenosterone, **g** 11-ketotestosterone, **h** progesterone,** i** 11β-OHP, **j** 11-DOC, **k** 16α-OHP, **l** 17α-OHP, **m** 11-deoxycortisol, **n** 21-deoxycortisol, **o** corticosterone, **p** aldosterone, **q** cortisone, **r** 18-OHB, **s** cortisol, and **t** 18-oxocortisol. Error bars indicate standard deviation (n = 5)

To identify the optimal steroid detection method, we compared the ion intensities of derivatized and non-derivatized steroids obtained by MALDI/MS, SALDI/MS, and ME-SALDI/MS. The results are presented in Fig. 3. Across all steroids tested, the derivatized steroids analyzed by ME-SALDI/MS exhibited the highest detection sensitivity. For most compounds, the ion intensities obtained by ME-SALDI/MS were more than twice those achieved with other ionization methods. For estrone, androstenedione, testosterone, DHT, adrenosterone, 11β-OHP, and 18-oxocortisol, ME-SALDI/MS of the derivatized steroids yielded more than a tenfold increase in ion intensity compared with conventional MALDI/MS of the derivatized steroids. Ion intensities increased by 11-fold for estrone, tenfold for androstenedione, 22-fold for testosterone, 15-fold for DHT, 11-fold for adrenosterone, 17-fold for 11β-OHP, and 52-fold for 18-oxocortisol. Among these, estrone, adrenosterone, 11β-OHP, and 18-oxocortisol are low-abundance steroid hormones with important biological roles. Estrone is an estrogen metabolite of estradiol. 11β-OHP is a progesterone-derived intermediate involved in the biosynthesis of mineralocorticoids and glucocorticoids. 18-oxocortisol is overproduced in patients with primary aldosteronism. Although these steroids exhibit relatively weak biological activity, they serve as important intermediates in steroidogenesis [27–29]. The enhanced sensitivity achieved in this study enables more reliable detection of these analytes and may provide new insights into disease-specific steroid metabolism.

When comparing the ion intensities of the non-derivatized steroids analyzed by SALDI/MS and ME-SALDI/MS, some steroids showed higher ion intensities in one method than in the other, depending on the compound. This finding indicates that the optimal ionization method for non-derivatized steroids is compound dependent. The highest ion intensities were obtained by combining GirT derivatization with ME-SALDI/MS, and, under the present experimental conditions, ME-SALDI/MS yielded higher ion intensities than SALDI/MS for all GirT derivatized steroids examined. In contrast, the relative ion intensities obtained by GirT-SALDI/MS and non-derivatized ME-SALDI/MS varied with steroid species. Collectively, these results suggest that the improved sensitivity reflects the combined effects of increased ionizability following GirT derivatization and more efficient desorption/ionization in ME-SALDI/MS. Accordingly, for non-derivatized steroids, the ionization method should be selected based on the target compound. The improved ion intensities observed in ME-SALDI/MS for derivatized steroids can be rationalized as follows: The desorption mechanism of SALDI is primarily thermal. Upon UV laser irradiation, the metal thin film with low thermal conductivity is rapidly and locally heated, promoting analyte desorption and ionization [24]. In contrast, MALDI involves laser absorption by an organic matrix with strong UV absorbance, and matrix ablation facilitates analyte desorption and ionization. The higher desorption/ionization efficiency observed for ME-SALDI compared with SALDI is attributed to the combined contributions of metal-assisted desorption/ionization on the Pt film and additional explosive desorption induced by matrix ablation in MALDI. The higher desorption/ionization efficiency of SALDI compared with MALDI may reflect the relatively high energy required for steroid desorption/ionization. In MALDI, matrix-mediated energy buffering reduces desorption/ionization efficiency, whereas in SALDI, more efficient ionization is enabled through localized thermal energy transfer. In the presence of a MALDI matrix during analysis, Na⁺ ions could also be desorbed from the sample, and gas-phase interactions between desorbed Na⁺ and neutral steroid molecules could contribute to the formation of sodium-adduct ions, [M + Na]⁺, from non-derivatized steroids. By integrating features of both SALDI and MALDI, ME-SALDI could enhance desorption dynamics and ion yield, although this effect depends on the steroid form. For non-derivatized steroids, sodium-adduct formation as [M + Na]⁺ is influenced by the physicochemical properties and structural features of each steroid. Therefore, enhanced desorption in ME-SALDI does not necessarily translate into improved ion yields over SALDI when sodium-adduct formation as [M + Na]⁺ does not proceed efficiently. In contrast, derivatization alters the ionization behavior of steroids and promotes molecular-ion formation as [M]⁺. This allows the enhanced desorption in ME-SALDI to contribute more effectively to increased ion yield.

These results clearly demonstrate that the choice of ionization method significantly influences detection sensitivity and ionization efficiency in steroid analysis. Moreover, the findings indicate that ME-SALDI/MS of derivatized steroids yields significantly higher ion signals than conventional methods, enabling more sensitive detection of trace-level steroids.

### MS imaging of porcine adrenal tissue sections

**Fig. 4:**
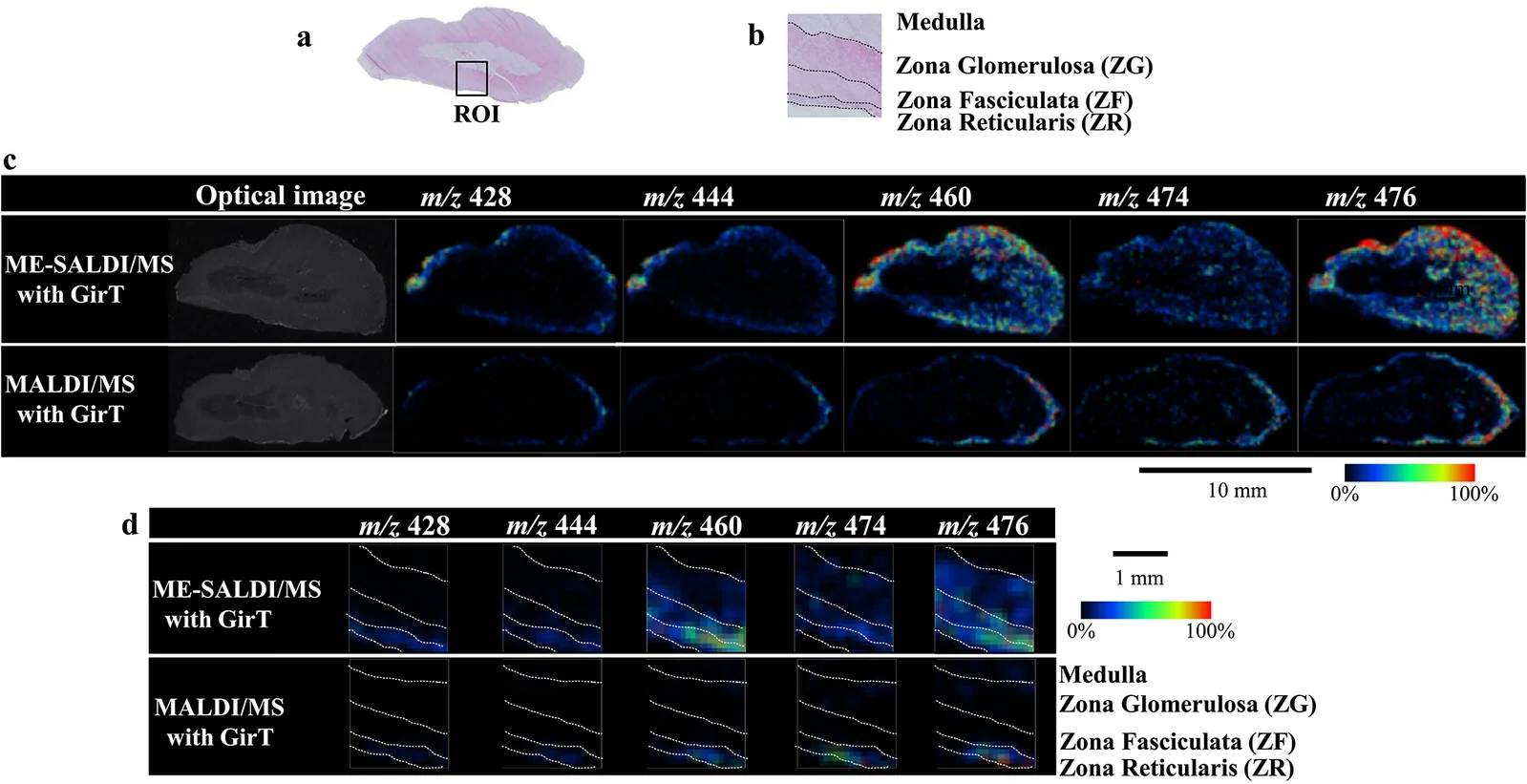
Images of the porcine adrenal tissue section: histological images of the hematoxylin- and eosin-stained frozen section showing **a** the whole tissue section and **b** the ROI (ZG: zona glomerulosa, ZF: zona fasciculata, ZR: zona reticularis). ME-SALDI and MALDI/MS images of derivatized steroids in porcine adrenal tissue sections:** c** the whole tissue section and** d** the ROI

The adrenal gland consists of two main regions: the inner medulla and the outer cortex. The adrenal cortex comprises three distinct layers—the zona glomerulosa, zona fasciculata, and zona reticularis—arranged from the outermost to the innermost region. Steroid hormones are synthesized within these cortical layers [25]. Figure 4a shows an HE-stained image of a porcine adrenal gland section, and Fig. 4b presents an enlarged view of the boxed area in Fig. 4a. The three-layered structure of the adrenal cortex is clearly visible. The same porcine adrenal tissue section was then analyzed by MALDI and ME-SALDI/MS imaging to visualize the spatial distribution of derivatized steroids. The distributions of the detected steroids are shown in Fig. 4c. The MS images indicate that the spatial distributions of the analytes were preserved during the solution-based GirT derivatization step, with no appreciable analyte redistribution. Several steroids have already been reported in the tissue. [18, 30, 31] This approach could therefore be useful for comprehensive steroid profiling, including the analysis of previously reported steroids.

In ME-SALDI/MS imaging, several derivatized steroids were detected as [M]^+^ ions. The detected steroids and their corresponding *m/z* values were as follows: progesterone (*m/z* 428.331); 16α-OHP, 11-DOC, and 17α-OHP (*m/z* 444.324); corticosterone and 11-deoxycortisol (*m/z* 460.317); aldosterone and cortisone (*m/z* 474.297); and 18-OHB and cortisol (*m/z* 476.312). For comparison, MALDI/MS imaging was also performed under the same conditions. Several derivatized steroids were detected as [M]^+^ ions, including progesterone (*m/z* 428.325); 16α-OHP, 11-DOC, and 17α-OHP (*m/z* 444.320); corticosterone and 11-deoxycortisol (*m/z* 460.319); aldosterone and cortisone (*m/z* 474.299); and 18-OHB and cortisol (*m/z* 476.314). In contrast, 11β-OHP and 21-deoxycortisol were not assigned because they were not detected by LC/MS analysis of steroid extracts from adrenal tissue. However, the overall detection sensitivity in MALDI/MS imaging was relatively low. These results demonstrate that ME-SALDI/MS imaging of derivatized steroids provides higher ion intensities and enables clearer visualization of steroid localization within the adrenal cortex than conventional MALDI/MS imaging.

In the zona glomerulosa, which regulates blood pressure and electrolyte balance, mineralocorticoids such as 11-DOC, 18-OHB, and aldosterone are synthesized. In the zona fasciculata, which controls immune responses and metabolism, glucocorticoids such as 11-deoxycortisol, corticosterone, cortisone, and cortisol are produced. In the zona reticularis, precursors of sex hormones, including 16α-OHP and 17α-OHP, are synthesized [25]. As shown in Fig. 4, all of these steroids were detected in the adrenal cortex. Although the three cortical layers were not clearly distinguishable in the MS images, some steroids may migrate across layers after biosynthesis. Nevertheless, their spatial distribution was clearly visualized with high sensitivity. Thus, ME-SALDI/MS imaging successfully captured the characteristic spatial localization of steroid biosynthesis within the adrenal cortex, providing valuable molecular insights into adrenal physiology and hormone regulation at the tissue level.

These results demonstrate that Pt thin film–based SALDI/MS and ME-SALDI/MS (Pt thin film combined with CHCA) provide a reproducible approach with improved ion yields for visualizing steroid distributions in biological tissues with minimal sample pretreatment. This method, which combines on-tissue GirT derivatization with ME-SALDI/MS imaging, provides a useful platform for biochemical imaging of steroids and could be extended to isomeric steroids and related compounds with low desorption and ionization efficiency.

## Conclusion

In this study, a novel analytical method for steroid detection with improved ion yields based on platinum (Pt) thin film–assisted SALDI/MS was developed. The Pt thin film enabled direct detection of non-derivatized steroids with excellent mass accuracy and reproducibility, demonstrating its strong ionization capability for neutral compounds. Furthermore, combining Pt thin film–based SALDI with the organic matrix CHCA in matrix-enhanced SALDI/MS (ME-SALDI/MS) markedly improved detection sensitivity, particularly for derivatized steroids. This synergistic effect likely arises from the integration of metal-assisted thermal desorption and matrix-assisted ablation.

Applying GirT derivatization and ME-SALDI/MS imaging to porcine adrenal gland tissue enabled visualization of the spatial distributions of multiple species of derivatized steroids with high clarity and sensitivity. The ME-SALDI/MS imaging results indicate that this method provides a sensitive platform for steroid analysis that is suitable for imaging trace-level steroids. Collectively, this work establishes an MS-based imaging methodology that combines sample pretreatment with high-sensitivity analysis of biological tissue sections.

## Data Availability

Data underlying the results presented in this paper are not publicly available but may be obtained from the authors upon reasonable request.
